# Editorial: Surgical treatment and perioperative organ protection for coronary heart disease and comorbid chronic diseases

**DOI:** 10.3389/fmed.2025.1752578

**Published:** 2025-12-08

**Authors:** Kui Zhang, Congcong Zhang, Yue Yuan, Jian Cao, Jiayang Wang

**Affiliations:** 1 Institute of Cardiac Surgery, Beijing An Zhen Hospital Capital Medical University, Beijing, China; 2 Department of Pathology and Pathophysiology, Beijing Anzhen Hospital, Capital Medical University, Beijing, China; 3 Department of Cardiology, The First Affiliated Hospital of Harbin Medical University, Haerbin, China; 4 Baylor College of Medicine, Houston, TX, United States

**Keywords:** comorbid chronic diseases, coronary heart disease, integrated management approaches, perioperative organ protection, surgical treatment

Data from the World Health Organization clearly indicates that cardiovascular diseases remain the leading cause of death globally (1). Among these, Coronary Heart Disease (CHD) patients with multiple chronic comorbidities exhibit significantly higher mortality, readmission rates, and long-term disability rates compared to those with CHD alone. This risk superposition is not merely arithmetic; it stems from complex interactions and vicious cycles of multiple pathophysiological processes. For instance, diabetes accelerates atherosclerotic progression and leads to diffuse vascular disease, increasing the technical difficulty of revascularization and compromising its long-term patency rates (2). Chronic kidney disease significantly elevates the risk of perioperative bleeding, thrombosis, and acute kidney injury by affecting coagulation, drug metabolism, and vascular calcification (3). The strong connection between the heart and the brain (the “heart–brain axis”) means that hemodynamic fluctuations during cardiac surgery can readily translate into cerebral hypoperfusion, leading to serious neurological complications such as postoperative cognitive dysfunction or stroke.

Therefore, for patients with CHD and comorbidities, the goal of surgical treatment extends far beyond successful revascularization. It resembles navigating a minefield filled with “traps,” where the core challenge lies in identifying, assessing, and protecting the function of other already affected or potentially vulnerable vital organs while performing the necessary cardiovascular intervention. This elevates perioperative organ protection from a supportive measure to a strategic cornerstone determining the overall success of the procedure and the patient's long-term outcome.

## 1 Dilemmas in current clinical practice and the evolution of concepts

Although the philosophy of “patient-centered care” is widely endorsed, specialized, fragmented healthcare delivery models often dominate daily practice. A cardiologist might focus on intervening in the coronary lesion while paying insufficient attention to the patient's renal function fluctuations; an endocrinologist may strive for precise glycemic control but underestimate the risk assessment associated with the stress of an impending cardiac surgery. This approach leads to isolated treatment decisions, failing to provide a comprehensive, coordinated management plan for patients with comorbidities.

Encouragingly, with the deepening of the concepts of “integrated heart–brain therapy” and “integrated heart–kidney therapy”, clinicians have begun to focus on integrated medical care. Integrated Care emphasizes treating the patient as a complete physiological, psychological, and social entity. Its core lies in breaking down the barriers between traditional internal medicine, surgery, and various subspecialties, establishing a demand-oriented, multidisciplinary team collaborative system. However, a significant divergence remains between conceptual consensus and the widespread, replicable implementation of clinical pathways. This gap stems from systemic deficiencies in knowledge frameworks, research support, and healthcare payment structures.

## 2 Key research directions and core pathways to breakthrough

To bridge this gap, we must undertake systematic research efforts in the following critical areas:

1. Deepening basic mechanistic research to elucidate the pathophysiological basis of comorbidity interactions. Our understanding of how CHD and various comorbidities interact at the molecular, cellular, and organ levels remains elementary. Future researches needs to explore deeper into shared signaling pathways (e.g., inflammation, oxidative stress, neurohormonal activation), metabolic disorders (e.g., insulin resistance, uremic toxins), and immune mechanisms involved in the pathogenesis and progression of comorbidities. For instance, investigating the “gut–heart axis” or specific inflammatory cytokines common to atherosclerosis and cognitive impairment may reveal new targets for “Simultaneous Treatment of the Heart and Gut” (4). This is the foundation for achieving precise prevention and targeted therapy.
2. Building precise perioperative risk assessment and early warning systems. Traditional risk prediction models (EuroSCORE II Score, STS Score), while widely validated, may have limited predictive power in comorbid populations. We need to take advantage of real-world big data, artificial intelligence, and machine learning algorithms, integrating multi-omics information, imaging characteristics, and dynamic physiological monitoring data to develop a new generation of dynamic risk prediction tools. This will not only enable the early identification and precise stratification of postoperative adverse events (acute kidney injury, stroke, death) but also provide quantitative evidence for personalized treatment decision-making (5).
3. Fostering coordinated innovation in surgical techniques and perioperative management strategies. Minimally invasive Coronary Artery Bypass Grafting (CABG), interventions for structural heart disease and other methods offer less invasive and faster recovery options for high-risk comorbid patients. However, technological innovation must go hand-in-hand with the optimization of perioperative management strategies, include refining organ protection strategies, individualizing anesthetic management, integrating evidence-based guidelines.
4. Constructing an integrated care model spanning the entire lifecycle. Surgery is merely a critical “node” in the patient's long journey of disease management. We must extend our vision to encompass preoperative optimization and long-term secondary prevention, rehabilitation, and health management post-surgery. This requires establishing a continuous care system traversing “community screening—preoperative assessment—surgical treatment—critical care—discharge practice planning—long-term follow-up”. Promoting cardiac rehabilitation, which integrates exercise training, nutritional guidance, psychological support, and smoking/alcohol cessation, is crucial for improving functional status, enhancing quality of life, and reducing the risk of recurrent events (6).

## 3 Call to action: collaborating to create a new era of integrated healthcare

Confronted with the complex, giant system of CHD and its comorbidities, no single discipline or technology can prevail alone. We stand at a crossroads demanding a paradigm shift in healthcare. The core of this transformation is a fundamental move from “treating diseases” to “treating the person,” from “fragmentation” to “integration.”

We issue a call to action:

1. To researchers. Embrace interdisciplinary collaboration actively, facilitate the translation of basic research into clinical practice, and dedicate efforts to solving key scientific problems in comorbidity management.
2. To clinicians. Proactively break down disciplinary barriers, participate actively in multidisciplinary teamwork, and implement the principles of Integrated Care in the treatment plan for every single patient.
3. To policymakers and healthcare administrators. Work toward building payment systems, performance metrics, and IT platforms that incentivize Integrated Care, providing the institutional backbone for this transformation.
4. To academic journals and professional societies. Continue to provide prioritized platforms for publishing such research, organize topical discussions, formulate consensus documents, and guide the academic direction.

The challenges are undoubtedly immense, but opportunities and hope coexist. Only through close global collaboration and relentless innovation can we translate scientific discoveries into tangible benefits for patients, successfully navigate the “bottleneck” of surgical treatment and perioperative management for patients with CHD and comorbidities, and collectively stride toward a new, more efficient, humane, and patient-centered era of Integrated Healthcare.
